# Health and Humanities and Social Sciences Professionals’ Perceptions Regarding the Teaching of the Effects of Racism in Medicine: Semistructured Interview Study

**DOI:** 10.2196/90084

**Published:** 2026-04-17

**Authors:** Mathilde Lambert, Rahmeth Radjack, Adil Mouhab, Malika Mansouri, Marie Rose Moro

**Affiliations:** 1 Université Paris Cité, 71 Avenue Edouard Vaillant, Boulogne-Billancourt (Hauts-de-Seine), 92100, France, 33 0176533179; 2 CESP-UVSQ, DevPsy, INSERM, Université Paris-Saclay, Villejuif, France; 3 Cochin Hospital, APHP, Maison de Solenn, Paris, France; 4 UTRPP Laboratory, Université Sorbonne Paris Nord, Villetaneuse, France

**Keywords:** racial discrimination, racism, Mediterranean syndrome, health inequalities, medical pedagogy, qualitative methods, health professionals, humanities and social sciences

## Abstract

**Background:**

In France, as in other countries, health disparities arise from multiple interacting factors, among which racism plays a significant role. Racism affects health through exposure to discrimination-related stress, environmental conditions, differential access to and quality of care, as well as representations and behaviors, some of which are rooted in the historical legacy of colonial medicine.

**Objective:**

This study aimed to explore the perspectives of health care professionals and researchers in the humanities and social sciences (HSS) on the core content areas to be defined and the corresponding learning objectives to be established for the development of a curriculum addressing racial discrimination in medicine for undergraduate medical education.

**Methods:**

Physicians, psychologists, and researchers in the HSS who had published on the medical care of people from racialized minorities and who had organized or participated in educational initiatives on this topic were recruited. Semistructured interviews were conducted and analyzed using reflexive thematic analysis.

**Results:**

This study is the first French study to bring together the experiences of French-speaking experts in this field to inform the development of educational content integrating medicine, psychology, and the HSS. A total of 20 participants were interviewed. Three main themes emerged: (1) teaching how to identify and dismantle stereotypes in clinical practice; (2) teaching the psychological effects of racism and its overall impact on health; and (3) teaching the risks associated with racialized care and so-called colorblind approaches. Participants emphasized the importance of addressing racial stereotypes specific to the French context, such as the “Mediterranean syndrome,” situating them within their historical background, and understanding the effects of discrimination on both physical and mental health. A key pedagogical challenge identified was finding a balance between acknowledging geographical or sociological specificities and avoiding culturalist interpretations that overemphasize context. All participants highlighted the necessity of such comprehensive education to ensure patient safety for all.

**Conclusions:**

Teaching about the effects of racial discrimination and racism on health should be framed as a core public health issue. Although racial discrimination is influenced by economic, political, and sociological factors that extend beyond the medical sphere, its impact on health is neither inevitable nor immutable and therefore warrants explicit educational attention. From a future perspective, the ongoing French debate regarding the feasibility of conducting epidemiological studies on racial health disparities needs to be addressed to further inform education, research, and policy.

## Introduction

The term racism, as defined by Camara Jones in a seminal article, refers to a conceptual framework encompassing 3 levels: institutionalized racism, personally mediated racism, and internalized racism [1]. Race is understood as a social construct that accurately captures the impacts of racism, given that racial classification has no legitimate biological basis [2]. Racial discrimination, as the effect or outcome of prejudice, refers to any unfavorable treatment of an individual based on these grounds, as well as any action that limits the opportunities of an individual or group due to characteristics such as race. In France, as in other countries where these phenomena have been documented, health disparities result from multiple interacting factors, with racism constituting a significant determinant.

First, the effects of racism and racial discrimination on health in general—and on mental health in particular—have been documented for several decades [3-8].

Second, challenging the genetic basis of racial categories and acknowledging that socioeconomic factors alone do not fully account for health disparities, a 2022 *Lancet* Commission report [9] examined the multiple pathways leading to poorer health outcomes among racialized minority populations. Stress responses associated with discrimination—including potential epigenetic changes that may be transmitted to subsequent generations—inequitable access to education, employment, healthy housing free from air pollution, green spaces, nutritious food, as well as the disproportionate health effects of climate change on minoritized populations, increase vulnerability to disease, coupled with higher occupational exposure risks and more limited access to timely diagnosis and appropriate treatment. These latter dimensions are particularly crucial for medical students to understand, as they will ultimately be key actors in addressing and reducing these inequities.

Such understanding requires multiple levels of analysis and dedicated educational content, especially since barriers to diagnosis and treatment are historically embedded, including within France’s colonial past. In France, the medical field has historically produced and perpetuated racial and racist stereotypes, traces of which remain observable in contemporary clinical practice. The French Defender of Rights has highlighted persistent obstacles in patient-provider communication, clinical management, and access to medications [10].

As early as the 20th century, Foucault [11] emphasized that the use of race introduces differential management of populations. Paradoxically, even if the French context is characterized by an official rejection of racial or ethnic categories, Fassin [12] has described the use of substitute categories that allow differences to be discussed without being explicitly named, a practice that may reinforce stigmatization and contribute to the construction of social identities. The resulting differentiated care—whether related to access to health care facilities [13], implicit biases leading to overdiagnosis or underdiagnosis of certain conditions [14], or the use of clinical formulas incorporating racial characteristics without scientific basis [15,16]—remains present in France and requires dismantling through multilevel interventions. A key challenge is ensuring the appropriate use of geographical and sociological specificities to enhance patient safety without reinforcing racialized assumptions.

Despite these challenges, only a limited number of courses addressing these issues are currently offered to French medical students [17], and they largely stem from individual initiatives, notably those developed over the past 3 decades by specialists in transcultural psychiatry in France. Their work has focused on access to professional interpretation services, shared cultural representations of care, consideration of migration trajectories when relevant, and the identity construction of children and adolescents navigating multiple spheres of belonging—processes that can be profoundly affected by racial discrimination.

With a view to improving this pedagogical approach in France, the objective of this study is therefore to explore the perspectives of health care professionals and scholars in the humanities and social sciences (HSS) on the core content areas to be defined and the corresponding learning objectives to be established for the development of a curriculum addressing racial discrimination in medicine for undergraduate medical education.

## Methods

### Study Design

A qualitative study was conducted from January to June 2024 using semistructured interviews and reflexive thematic analysis (TA) and was designed and reported in accordance with the COREQ (Consolidated Criteria for Reporting Qualitative Research) guidelines. This approach was appropriate for exploring how health professionals and experts in the HSS understand and teach the effects of racism in medicine.

### Participant Characteristics

Inclusion criteria were being a French-speaking professional specializing in medical issues and having worked on matters of racial discrimination in medicine, that is, having published on health care for racial minority populations and having organized or participated in related training or courses.

The study focuses on the French context by interviewing individuals with in-depth knowledge of this setting. Most participants were therefore French. However, the still-emerging nature of medical education in France in this area supported the inclusion of several francophone professionals from other countries, with the aim of fostering the exchange of best practices. Purposive sampling ensured diversity across profession, epistemology, practice setting, country, age, and gender to maximize qualitative validity. Participants included professors of medicine or psychology, teacher-researchers in the HSS with PhDs, and health care professionals with advanced academic qualifications in HSS. Several had established university programs on transcultural issues and health inequalities, led courses on discrimination, or coordinated medical curricula integrating these topics. Medical education on these topics must be conceived as inherently interdisciplinary and draw on multiple methodological approaches. This rationale motivated the inclusion, in this study, of professionals from diverse disciplinary backgrounds.

### Data Generation

Participants were contacted via email by the primary investigator (ML). Semistructured interviews were conducted by ML from March to July 2024, either in person or remotely for those outside the Île-de-France region, lasting approximately 60‐90 minutes. Verbal consent was audio-recorded. Out of 31 contacted individuals, 3 declined due to availability, 8 did not respond, and 20 agreed to participate.

Interview guides (Multimedia Appendix 1) were tailored to the participants’ professional profiles, grouped into 3 categories: physicians, psychologists, and teacher-researchers in HSS. Topics included racial discrimination in health care, educational practices and formats, physical and psychological impacts of racism, awareness-raising strategies, and the contribution of HSS to teaching. Audio recordings were transcribed verbatim.

### Data Analysis

A reflexive TA was conducted using the 6-step framework by Braun and Clarke [18-20]: familiarization with data, generation of initial codes, development and review of subthemes and themes, refinement of themes, and reporting. Transcripts were analyzed using NVivo 14 (Lumivero). Initial inductive coding was performed sequentially by ML. The study was based on a logic of interpretive sufficiency, with the corpus being deemed adequate when it supported an in-depth and coherent reflexive analysis of the phenomenon under study.

Codes were iteratively grouped into subthemes and themes through multiple revisions, resulting in an initial thematic framework. A cross-sectional analysis was conducted independently by ML and MM, that is, a precise and systematic comparison and analysis of themes and subthemes in relation to codes and significant verbatim quotes. It was followed by iterative discussions with ML, MM, RR, and MRM to ensure triangulation and reflexivity. Disagreements were pooled and discussed each time they arose, with back-and-forth exchanges of arguments and a collective decision made. This process led to consensus on final themes, supporting rigor and credibility. The analysis was data-driven, with themes identified at a semantic level and developed iteratively from description to interpretation [21]. Reflexive TA procedures reflect the values of a qualitative paradigm, centering researcher subjectivity, organic and recursive coding processes, and the importance of deep reflection on, and engagement with, data [20].

### Team Reflexivity

The positionality of the research team may have influenced data collection and analysis. The interviewer (ML), a physician perceived as belonging to the French majority group, could have shaped interview dynamics and participants’ responses. In accordance with French regulations, no systematic data on participants’ ethnic or self-identified group affiliations were collected, though some participants voluntarily shared aspects of their background. This limitation may affect the interpretation regarding participants’ positioning relative to the majority group.

The authors of this article have academic and professional training in France, with diverse cultural backgrounds and family histories marked by migration, which adds value and richness to the perspective. The authors are trained in transcultural psychiatry, which involves the complementary use of data from humanities disciplines such as anthropology, history, and sociology. One of the key concepts of transcultural psychiatry is decentering, which allows one to put oneself in another’s shoes. Qualitative data triangulation spaces were regularly used to enable participants to become aware of their own feelings and biases toward the research topic.

The authors’ engagement with antiracism issues may have influenced the interpretation of findings as tools for improving teaching practices. Reflexive triangulation was used among team members with diverse professional backgrounds, experiences, and age groups. Collective discussions, including a monthly doctoral seminar, supported ongoing reflexivity. These reflective discussions led to the renaming of themes that were potentially too descriptive and insufficiently analyzed, refocusing them on their pedagogical implications in relation to the study’s objective. For example, the first theme, “Pain and Mind and Mind-Body Dualism: The Invocation of Stereotypes,” was renamed “Teaching How to Identify and Deconstruct Stereotypes in Clinical Practice.” Similarly, the theme “Inequalities in Access, Epidemiology, and Differentiated Care” was removed after discussion and merged with the other 3 themes because it did not, on its own, address the objective.

Cultural countertransference [22] was explicitly considered as an analytic resource, allowing engagement with participants’ experiences of alterity while avoiding culturalist or exoticizing interpretations.

### Ethical Considerations

Participants were contacted by email and informed about the study objectives, interview procedures, and confidentiality measures. Oral consent was obtained for both participation and the use of interview data in publications. Each participant was assigned a random alphanumeric code to ensure confidentiality. Interviews were analyzed by the research team, and data were securely stored on a password-protected computer managed by the primary investigator (ML). Data were retained for 1 year and subsequently destroyed. The study was approved by the INSERM (French National Institute for Health and Medical Research) ethics committee in March 2024 (CEEI/IRB No 24‐1068) and conducted in accordance with the ethical principles of the Declaration of Helsinki.

## Results

### Overview

The study included 20 participants from diverse professional backgrounds, including medical doctors, psychologists, and teachers in the HSS (Table 1).

**Table 1.** Characteristics of the study participants (N=20).

Participants	Gender	Profession	Country
ID01, M	Women	Child and adolescent psychiatrist	France
ID02, nurse and HSS	Men	Nurse and Anthropologist	France
ID03, M	Women	Psychiatrist	France
ID04, M	Men	Dermatologist	France
ID05, M	Women	Pediatrician	France
ID06, HSS	Women	Sociologist	France and United States
ID07, M	Women	Gynecologist-Obstetrician	France
ID08, M	Men	Child and adolescent psychiatrist	Canada
ID09, P	Men	Psychologist	Canada
ID10, HSS	Women	Historian	France
ID11, M	Men	General practitioner	France
ID12, M	Women	Child and adolescent psychiatrist	France
ID13, M	Women	Psychiatrist	Canada
ID14, M	Men	Internist	Switzerland
ID15, HSS	Men	Anthropologist	United States
ID16, HSS	Men	Sociologist	Belgium
ID17, midwife and HSS	Women	Midwife and Sociologist	France
ID18, nurse and HSS	Women	Nurse and Sociologist	France
ID19, P	Women	Psychologist	France
ID20, P	Women	Psychologist	France

This multidisciplinary sample enabled the inclusion of complementary perspectives on the topic. Health care professionals contributed insights grounded in clinical and hospital practice, as well as in their interactions with students. These perspectives both complemented and contrasted with psychological approaches, which tend to emphasize how students receive information and the potential psychological effects of discrimination. The inclusion of the HSS was also essential, as these disciplines provide conceptual frameworks for understanding the social and historical dimensions of medicine, thereby supporting students’ comprehension of the broader context in which their training takes place.

For clarity, participants’ professional roles are identified in verbatim quotations as follows: medical doctor (M), psychologist (P), and humanities and social sciences teacher (HSS).

Three main themes emerged (Table 2): (1) teaching how to identify and dismantle stereotypes in clinical practice; (2) teaching the psychological effects of racism and its overall impact on health; and (3) teaching the risks associated with racialized care and so-called colorblind approaches.

Regarding the terms used in the results, it is worth recalling that although race is widely recognized as a social construct rather than a biological category, observable phenotypic traits and patterns of genetic ancestry may carry clinically relevant information in specific contexts.

Phenotype can function as a provisional clinical cue—particularly in contexts where genetic ancestry, environmental exposures, or structural inequities correlate with differential disease prevalence or treatment response. However, its use must remain probabilistic, context-dependent, and critically examined. Treating race as a biological essence risks reifying socially constructed categories and perpetuating bias; conversely, ignoring how socially mediated patterns of ancestry and embodiment affect health outcomes may obscure clinically relevant risk stratification.

Therefore, the pedagogical task is to train clinicians to distinguish among race as a social category, ancestry as a population-level genetic construct, and phenotype as an observable set of traits—while foregrounding the role of structural determinants of health. Responsible clinical reasoning requires acknowledging human variation without naturalizing racial categories or reinforcing deterministic assumptions.

**Table 2.** Themes and subthemes found.

Themes	Subthemes
Teaching how to identify and dismantle stereotypes in clinical practice	Mediterranean syndrome and the hierarchy of pain Ignoring context or overemphasizing it: two risks of misdiagnosis Learning about illness-related behaviors through history to move beyond stereotypes
Teaching the psychological effects of racism and its overall impact on health	Understanding the impact of racial discrimination on health and ensuring patient safety Understanding the consequences of assimilation to support patients’ processes of subjectivation
Teaching the risks associated with racialized care and so-called colorblind approaches	The pitfall of culturalism The question of differentiated formulas or protocols Explaining the concept of sociological race and accounting for health-relevant ethnic differences

### Teaching How to Identify and Dismantle Stereotypes in Clinical Practice

Mediterranean syndrome and representations of pain crystallize racist stereotypes in care, leading to diagnostic errors, including either a culturalist consideration of the context or ignorance of this context and rejection of the associated psychological causes. All of this creates a risk of misdiagnosis and medical error.

#### Mediterranean Syndrome and the Hierarchy of Pain

Racial stereotypes were highlighted by most participants, with a particular focus on pain, both in France, including in La Réunion, as well as in Switzerland and Canada:

Yes, Comorians and Mahorais are compared to other inhabitants. There’s a syndrome, the fatigue syndrome. We'll tell you, the Mahorais are tired.

[ID02, nurse and HSS]

Mediterranean syndrome was central to the discussions and was described as an archetypal racist medical stereotype in Europe. This racist pseudosyndrome refers to the inadequate clinical management of symptoms perceived as exaggerated, based on the essentialization of individuals according to assigned racial identities and associated prejudices, often rooted in colonial medicine or the history of slavery. It has been identified as a source of medical error and potentially preventable harm. In France, this phenomenon has primarily affected individuals identified as Arab, Black, or even Roma, while in Switzerland, similar mechanisms have historically targeted Italian patients under the label of “Transalpine syndrome.”

In contrast, in North America, participants referred to stereotypes portraying Black patients as having a higher resistance to pain. Referring to the example of sick leave duration being adjusted according to racial prejudice, one participant emphasized the need to avoid differential treatment, which otherwise leads to racial and social discrimination.

The doctor doesn’t have to ask himself that question, he just has to hand out a piece of paper. The people who live in Montréal-Nord can barely read or don’t know the system. Above all, they are already ashamed and don’t want to ask for more. And so these are people who are going to go to hospital, receive care, and will never have time off or rest. So these are racial issues, it’s racism, it’s racial discrimination.

[ID09, P]

A participant from the United States highlighted that the sexist dimension of historical stereotypes related to pain is often compounded, reinforcing the dehumanization and hierarchization of patients. He referred to the notion of white vulnerability as a mirror image of prejudice that leads health care professionals to prioritize comfort and pain management for White patients.

Deirdre Cooper Owens, a historian, has clearly shown that there is a whole historical tradition that can be traced back to the beginning of the 19th century, around black women who are capable of giving birth without pain and who are naturally very fertile. There’s also the whole issue of dehumanization behind it, white vulnerability, the fact that more attention is paid to treating pain.

[ID06, HSS]

#### Ignoring Context or Overemphasizing It: Two Risks of Misdiagnosis

Two types of diagnostic error related to patient context were identified. Participants described, on the one hand, the risk of ignoring contextual factors, including a lack of awareness of certain geographical or cultural medical specificities. On the other hand, they highlighted the risk of overemphasizing context, leading to culturalism and diagnostic error, described by one participant as “the overshadowing*”* (ID04, M)

Overemphasis on context was associated with an alleged knowledge of culturally specific signs that obscured standard diagnostic reasoning.

The psychiatrist tells me, this is a rite of passage. All teenage girls go through some sort of djinn crisis during adolescence. She came back a few days later, a young girl who was really decompensating. There was nothing cultural about it, especially as nothing could lead us to think of a cultural aspect.

[ID02, nurse and HSS]

Another participant provided the example of a patient whose traumatic experience had resulted in severe physical suffering. She expressed indignation at the failure to consider the psychological context and at the immediate attribution of symptoms to “Mediterranean syndrome” by physicians who, in the absence of organic findings, resorted to racist prejudices.

#### Learning About Illness-Related Behaviors Through History to Move Beyond Stereotypes

Participants emphasized the relevance of teaching the history of science to contextualize the emergence of certain data rooted in the racist views of scientists, particularly during the colonial period. One participant referred to the notion of a North-South gradient in pain expression.

There was a period when it was politically correct, and there were even sociologists and anthropologists to corroborate that there was indeed a gradient in the expression of pain from North to South, that people shouted louder and louder when they were in pain. Then we realized that these were sociologists who had simply endorsed the racism of the doctors they were observing, that they were simply in the process of scientifically sociologising racist comments.

[ID11, M]

For this participant, focusing on illness-related behaviors allows clinicians to address individual characteristics in care delivery, thereby avoiding stereotypical reasoning and reducing the risk of misdiagnosis.

Is it the way in which, when we perceive an internal disturbance, we approach the medical profession? And what words do we use, how do we look, what questions do we ask? This approach to illness gives rise to listening skills. Because doctors need to be trained in how to deal with illness in order to react and avoid stereotypes.

[ID11, M]

### Teaching the Psychological Effects of Racism and Its Overall Impact on Health

The effects of racism on the psyche were described as multiple, ranging from impacts on subjectivation to persistent doubt regarding the legitimacy of experiences perceived as discrimination, leading to ongoing cultural insecurity.

#### Understanding the Impact of Racial Discrimination on Health and Ensuring Patient Safety

The psychological impact of racial discrimination was described as well documented in American studies, particularly with regard to microaggressions and more acute traumatic experiences.

The way I teach this is that I talk about cumulative trauma. The example I give is the glass of water you hold. You hold it for 10 seconds without worrying. After a minute, you start to feel a bit unwell. After 5‐10 minutes, it becomes very unpleasant. And then I mention stress, which has biological consequences.

[ID13, M]

Several participants referred to epidemiological studies, both North American and French, to describe the effects of racism on health, particularly mental health. These effects were sometimes described as being insufficiently recognized, including by those who experience them. The notion of cultural safety in care was emphasized, with participants warning of serious consequences if it is not ensured.

It means providing healthcare that is culturally safe and respectful of communities.

[ID13, M]

One participant addressed racism mediated through the gaze, referring to the concept of the racial epidermal schema developed by Caribbean psychiatrist Frantz Fanon, and emphasized the need to educate the clinical gaze in order to improve therapeutic relationships and counter racial prejudice.

He talks about the racial epidermal pattern, which means that in the lived experience of black people, they are victims of racism through the gaze. And that’s something we don’t work on enough in medicine, the gaze is never neutral, it’s part of a personal, medical and colonial history. And Fanon says, we have to educate our gaze, listen to classical music, operas, we have to read...

[ID03, M]

Capturing changes in the gaze before and after training was described as a potential tool for evaluating shifts in students’ subjectivity during teaching. Several participants also highlighted racial bias within the therapeutic relationship, including difficulties being taken seriously, the perceived obligation to dress or behave in certain ways to be heard, and the tendency for clinicians to rely on stereotypical interpretive frameworks in contexts of hospital crisis.

There’s such a crisis that there’s not much guarantee that we’ll be well looked after. But there’s still a bias at play, a kind of tacit reconduction, an acceptance of this kind of imposition of a line of reading that continues to work.

[ID20, P]

#### Understanding the Consequences of Assimilation to Support Patients’ Processes of Subjectivation

Regardless of their profession, some professionals seemed very aware of the psychological risks posed by assimilation and of the need to teach it to medical students through the humanities and psychology, as well as to raise awareness of it in practice.

One participant, who was both a health care professional and a researcher in the humanities, recounted an experience in which a North African health care worker laughed at racist jokes as a form of internalized racism and conformity to the norms of certain team environments.

I remember a surgeon who liked to say that patients from the Maghreb inevitably had a lot more pain than others because they were wimps and had Mediterranean syndrome. They used to say it to the staff, and even the North Africans laughed. When you’re a North African carer and you’re laughing at that, I understand that it doesn’t make sense to you in the end. And who were convinced that it was something that existed. That means we’re almost assimilating colonial thinking.

[ID02, nurse and HSS]

Frantz Fanon was again referenced, particularly his distinction between assimilation and integration, and his description of assimilation as a deprivation of self in order to resemble the other.

Fanon said that to be assimilated is the prefix A which is privative. He said that to be assimilated is to deprive oneself of a part of oneself or one’s entirety in order to become similar to the other. And he contrasted assimilation with integration. Integration means coming with what you are, taking the best of the other and incorporating it into yourself. That’s interculturality.

[ID02, nurse and HSS]

Assimilation was also discussed as a way of being in the world. One participant referred to the concept of double consciousness theorized by WEB Du Bois in 1903 in *The Souls of Black Folk*, describing the tension between imposed stereotypes and the constant effort to counter them.

This question of adaptability, psychologically, there’s a charge of always adapting to the other. You’re constantly thinking, consciously or unconsciously, about the stereotypes held about the group to which you’re perceived to belong. And there’s the action of constantly trying to thwart them in everyday life or, on the contrary, to overplay them. And once again, it’s a mask to try and hide behind, because in the end, showing yourself is perceived by these people as a source of danger. So it’s always black skin, white mask.

[ID19, P]

The contradictory injunctions related to belonging to the French collective were described as contributing to identification disorders that may become extreme and lead to forms of loss of identity anchors.

The effects are permanent illegitimisation of various kinds. Racialised people in French society are constantly reassigned to a specific position. This produces a form of identity crisis, let’s say, a disturbance in identification that can be extremely intense, an intimate experience of an omnipresent contradictory double injunction.

[ID20, P]

The effects of racism on the psyche were further described as phenomenological, particularly with regard to whether individuals’ identities are recognized within the therapeutic relationship. Nonrecognition was described as a form of violence and disqualification that is difficult to overcome.

Not taking the other person’s words into account in their own language, and therefore saying to them, your words are symmetrical, I’m going to treat you, you’re my fellow human being and your language is as good as mine. Really, when everything is done to put you in a position where the other person is less legitimate, it has a major impact on people’s self-esteem.

[ID12, M]

This participant further discussed, particularly in relation to children, the dissociation between cognitive and affective dimensions that such experiences may require, leading to increased vulnerability. Drawing on Georges Devereux’s concept from *La renonciation à l’identité. Défense contre l’anéantissement* (*The Renunciation of Identity: Defense Against Annihilation*), she emphasized the importance of recognition in enabling individuals to mobilize their full resources.

When all your affiliations are disqualified, it’s only the cognitive that can be put forward and that’s very vulnerable, not just for children. These are things we might call intersubjective. The more symmetrical the relationship, the more we can talk to each other, the more we respect the principle of psychological universality, the more likely it is that the other person will be able to use all his or her resources, and not put him or herself in a position of effacement or powerlessness, all things that do a lot of harm to the person, of renouncing his or her identity.

[ID12, M]

### Teaching the Risks Associated With Racialized Care and So-Called Colorblind Approaches

The issue of racialized care was described along 2 main lines. First, participants highlighted the persistent misuse of racial criteria based on stereotypes inherited from colonial medicine and slavery. Second, they emphasized the underrepresentation, in medical textbooks, research, and training curricula, of what they referred to as geographical minorities, as well as of specific clinical features that require consideration in order to provide effective care.

#### The Pitfall of Culturalism

Several participants, mainly HSS professionals, questioned the use of the term culture, noting that its meaning varied across contexts and countries. On the one hand, culture could be understood as a useful concept for engaging with patients’ representations of illness and ways of thinking about disease. On the other hand, participants warned that it could also be mobilized in a fixist and categorical manner that limits clinical reasoning and reinforces stereotypes. This latter use, referred to as culturalism, was described as an obstacle to care and a source of racial discrimination.

How do we sometimes refer to certain individuals, reduce their identity to so-called cultural stereotypes, on their way of thinking, their way of behaving. There are a certain number of representations that are attached to these individuals and which then condition the organization of a whole care system around these characteristics that do not correspond to the personality of the individual.

[ID06, HSS]

Referring to fieldwork conducted in the United States, the same participant described differences in care structures, including community-based services, in which health care providers were not always aware of their own stereotypes associated with particular communities. These representations were described as having direct consequences for the organization of care.

All this cultural organization also sometimes referred to very fixed categories, which we tried to develop in a different way and to differentiate from biologism, and which also referred to a whole strategy of representation which, itself, was very fixist and which again reduced the individual to boxes. We need to understand the social condition, the trajectories and the representations that these patients have of themselves and their families in order to reach out to them and improve care.

[ID06, HSS]

One participant described warning students against the overuse of the notion of culture, encouraging them to reflect on their own cultural frameworks and to mobilize cultural considerations only when sufficient time is available to explore patients’ representations. Otherwise, there is a risk of reinforcing culturalism.

I’d say be careful with the notion of culture, because when you say African culture, North African culture, it doesn’t really mean anything. Of course, you have to be taught to talk about yourself, talk about your body, express your feelings or not express them, hide them, and so on. If you don’t have the time to touch it, don’t touch it at all, forbid yourself to consider it in cultural terms. If you have the time, go and ask each of them, in fact, how do you want to give birth? Where do you get the urge to give birth like that, etc.? So go ahead, but really go ahead and take your time. And sometimes expect to be very disconcerted by the answers, because you’ve stereotyped something for yourself.

[ID17, midwife and HSS]

#### The Question of Differentiated Formulas or Protocols

The issue of differentiated clinical formulas or protocols was described as particularly salient in the United States. Several participants, combining transdisciplinary perspectives with physicians and HSS teachers, denounced the racist nature of the use of racial criteria without scientific basis, emphasizing the serious consequences for patient care.

There’s the story of glomerular filtration rate in nephrology. They noticed that from time to time African-American dialysis patients had high creatinine, and rather than blaming it on renal failure they said it was because they had a large muscle mass. They stacked up a series of clichés like that and ended up with this. We realized that taking racial criteria into account when calculating the formula made a difference to the management of early renal failure in African-Americans. I tried to dig deeper, I tried to go back to, shall we say, original sin. It’s impossible to find, really. There aren’t really any studies, any sort of concrete data, just a posteriori studies.

[ID04, M]

The participant further described how post hoc analyses based on racial categories were used to generate generalized conclusions and algorithms, despite their methodological limitations. In addition to nephrology, similar practices were reported in other specialties, including algorithms related to cesarean sections, fever, and other routine clinical decisions.

Another participant with research experience in the United States highlighted the routine use of ethnicity categories in patient forms through what was described as heterodefinition (ID06, HSS), with a limited number of categories primarily designed for administrative purposes. These categories were then applied mechanically in clinical contexts, often under the assumption of improving efficiency.

In the United States, we are so close to these ethno-racial categories that they are no longer questioned at all. And so we slide from a category that sometimes means something cultural, that sometimes means something social, as I was saying about the proxy, the social race, and that sometimes also means something biological.

[ID06, HSS]

The same participant noted that drug adaptations and dosage adjustments were often based on hearsay inherited from broader systems of representation related to stereotypes of hypermasculinity and physical strength, rather than on formal medical training.

I would refer you to a whole body of literature on the sociology of the body, the history of the body, to understand how, in medicine and psychiatry, we have been able to define care protocols that are more or less informal and different.

[ID06, HSS]

A critical approach to complementary examinations, particularly equations incorporating ethnic correction factors, was recommended to students by another participant, who emphasized the importance of documenting patient histories, noting that subsequent clinical decisions depend heavily on how cases are described.

The next colleague’s attitude will depend on the terms used.

[ID14, M]

In parallel, one participant working in Canada described efforts to take skin color into account in order to document discrimination in health care and identify the increased prevalence of certain diseases. The objective was to improve public health by drawing on community experiences and improving outreach and care.

Explaining the concept of sociological race and accounting for health-relevant ethnic differences

Training was described as addressing, first, the different meanings of the term race in medicine: historically as a biologized category, and sociologically as an assigned race whose effects must be recognized in order to be dismantled. Second, participants emphasized the need to address the specific medical needs of certain communities within the same geographical area.

One participant highlighted the mismatch between fixed phenotypical categories and the diversity of lived situations, which can lead to erroneous causal reasoning in diagnosis and treatment.

It’s true that it’s complicated for students. That’s why, when we talk about essentialism, we tend to associate a racial category, which is a reduction of what we observe, with a phenotype without understanding the complexity of the phenomenon. And it’s when we naturalize the category with reality, when we imbricate the two, that we create slippages and that these slippages can potentially have the effect of over-investing and over-producing causalities that in fact don’t exist.

[ID06,HSS]

Dermatology was cited as an example of the type of knowledge required to provide care that accounts for individual characteristics. One dermatologist participant challenged the idea that dermatology on Black skin is inherently difficult, noting that while specific features exist, they are easy to learn when training includes representations of diverse skin types.

The example of ethnic pseudoneutropenia among individuals from Africa or the Middle East was also discussed, particularly regarding its implications for the prescription of medications such as clozapine in psychiatry or for chemotherapy dosing.

To be on the safe side, we’re going to lower doses. In the United States, for example, it’s well known that breast cancer has a poor prognosis in African-American women, mainly because of lower chemotherapy doses.

[ID04, M]

Several participants pointed to insufficient training on health issues specific to certain communities, including in dermatology and psychiatry. This lack of training was associated with the overrepresentation of racialized communities in psychiatric services, particularly for psychotic disorders. Participants emphasized that Western medical training was historically designed to treat White populations, a limitation sometimes summarized by the expression “racism, not race,” highlighting the health consequences of racism itself.

We are ill-equipped to understand how certain psychiatric pathologies manifest themselves in certain communities. Because medical training has been based on treating white people. In psychiatry, we apply the DSM as if it had been made for everyone, whereas in Montreal, as I say, people from racialized communities are over-represented in our institutions, particularly when it comes to psychotic disorders.

[ID13, M]

## Discussion

### Principal Findings

The stated objective of this study was to identify the core content areas to be defined and the corresponding learning objectives to be established for the development of a curriculum addressing racial discrimination in medicine for undergraduate medical education.

A recurring challenge in medical education on these issues is avoiding culturalism, and our findings offer several insights in this regard. Specifically, the results underscore the importance of helping students recognize racial stereotypes and learn how to challenge them, understand the psychological burden of racial discrimination, and remain attentive to relevant geographical and sociological factors without essentializing patients or increasing the risk of misdiagnosis.

Several findings are consistent with existing literature, including the emphasis on hierarchies of pain perception [23,24], the risk of diagnostic error or misdiagnosis [25], and the dangers associated with clinical formulas or protocols differentiated on the basis of presumed ethnicity [26,27]. However, our study also highlights specific features that merit further discussion. Notably, likely due to the high proportion of psychologists and psychiatrists among participants, the psychological impact of racism on care and on identity emerged as a central theme. This psychological dimension is closely linked to the specificity of the “Mediterranean syndrome” in France, which is rooted in colonial history and requires critical examination to be effectively dismantled in both clinical practice and professional representations. Another key finding concerns the pedagogical challenge of enabling students to distinguish between medically relevant contextual knowledge and the continuation of culturalist reasoning that leads to unsafe, racialized care.

The aim is to provide future physicians with a form of symbolic authorization to introduce nuance into their clinical reasoning. In line with our previous work [17], which highlighted the importance of teaching students to reflect on countertransference in caregiving relationships, these results suggest that once such reflexive work is initiated, information about racism as a risk factor for health is more readily integrated into everyday clinical practice.

### Pain, Mediterranean Syndrome, and Their Historical and Psychological Dimensions

Racialization can be defined as the process by which race is socially constructed to enable the domination of one group over another, leading societies to view races as real, different, and unequal across social, economic, and political domains [1]. According to the definition of the Human Rights Commission of Ontario in 2005, “Racialized people” refers to people seen as belonging to racialized minorities and are people who could be perceived as being socially different from, for example, the racial or ethnic majority. The word “racialized” stresses the fact that race is neither biological nor objective but is a concept that is societal in origin [28].

The use of racialized categories in medicine reinforces the risk of culturalism in care—that is, attributing fixed and naturalized characteristics to presumed patient cultures, homogenizing groups while obscuring social, historical, political, and structural determinants of health. One consequence of racialization, and a paradigmatic example of how stereotypes can bias the therapeutic relationship, concerns pain management, a symptom that is both physical and psychological. Racism, sexism, and stigma toward mental health conditions intersect in the so-called “Mediterranean syndrome,” a term still used or implicitly invoked in French hospitals [29]. A growing body of French academic literature and media reports [29-31] has documented the seriousness of this prejudice, which often operates implicitly and is inherited from colonial medical traditions [32]. This issue has also been formally highlighted by the French Defender of Rights [10]. Such institutional recognition supports framing this phenomenon as a public health concern.

Our findings reveal the pervasive anxiety among patients about whether they will be taken seriously in emergency settings and suggest that this stereotype intersects with broader prejudices toward patients presenting physical symptoms without identifiable lesions or clear diagnoses, commonly categorized as functional or somatic symptom disorders [33]. These findings resonate with the work of Frantz Fanon in 1952 in Le syndrome nord-africain (The North African syndrome) [34,35], who emphasized the role of psychological suffering in physical symptoms among North African workers and highlighted how racialization of the body produces specific forms of alienation and distress. Participants expressed strong concern about clinical situations in which the psychological context was ignored and symptoms were immediately attributed to “Mediterranean syndrome,” reflecting the activation of racist assumptions in the absence of organic findings.

Drawing on the work of the phenomenological philosopher Merleau-Ponty [36], who emphasized that individuals engage with the world through their bodies, Frantz Fanon described the impact of the “White gaze,” which objectifies Black individuals by reducing them to their bodies—and ultimately to their skin—what he termed the “racial epidermal schema” [37]. According to Fanon, this imposed separation between body and subjectivity, developed as a response to racism, can generate both physical and psychological distress, including feelings of alienation and disruption of one’s body schema. These concepts resonate with our findings on the dissociation between affective and cognitive dimensions of care that can arise when patients’ identities and lived experiences are denied. Such dissociation may negatively affect psychological development and health by prioritizing cognitive explanations while fostering mistrust toward the expression of emotions. This highlights an often-overlooked link between 2 forms of bias in health care: the belief that suffering is only legitimate when it is visible, measurable, or objectifiable. When pain does not fit these criteria, it may be dismissed as exaggerated, feigned, or “merely psychological.” Framing symptoms as “cultural” or “all in the patient’s head” effectively invalidates suffering, including suffering related to experiences of racism. Even when overt accusations of simulation are absent, the systematic relativization of physical symptoms—particularly among racialized patients—risks not only serious diagnostic errors but also the erasure of the psychological meaning of these symptoms. Building on Fanon work and that of Gayatri Spivak, Maude Ludot, a child psychiatrist and researcher on somatoform disorders in adolescents, argues that somatic symptoms may function as expressions of experiences that cannot otherwise be articulated, particularly experiences of discrimination [38]. When such experiences are not heard or acknowledged, they may instead be expressed through physical symptoms later described as “medically unexplained.”

Research increasingly confirms the existence of a close link between experiences of racial discrimination and psychosomatic disorders, with some data also suggesting that these effects may be amplified in women [39]. From an educational and clinical perspective, it would be appropriate to develop a module on somatic and somatoform disorders linked to racial discrimination, in line with the concept of “embodiment of discrimination” [40], which aims to highlight how social processes (colonial heritage, microaggressions, denial of competence, etc) translate into concrete alterations of the body and the relationship to the body, involving psychological effects as well.

“Mediterranean syndrome” also stems from a misunderstanding between the legitimate need to consider so-called cultural factors when appropriate—particularly in the context of mental health care—and a form of culturalism that turns these factors into a generalized, essentialized, and ready-made explanatory framework, applied indiscriminately to patients without any knowledge of their individual affiliations.

### Between Culturalism and Colorblindness: Identifying Medically Relevant Factors

One key concept emerging from both Fanon work and our findings is that of situational diagnosis, which involves systematically considering environmental factors in clinical assessment. While contemporary medicine increasingly acknowledges social determinants of health, racism itself is rarely treated as a risk factor in medical histories, despite substantial evidence suggesting that racism—not race—is the relevant determinant of adverse health outcomes [41,42]. This distinction is essential for medical education.

Students often struggle to differentiate between the legitimate use of certain geographical, sociological, or phenotypic information and the pitfalls of culturalism and racialized care. Our findings suggest addressing this challenge through 3 complementary approaches.

First, students should be taught a limited number of clinically relevant contextual features. Dermatology education and the importance of representing all skin tones [43-45]; so-called ethnic pseudoneutropenia whose benign nature, if not recognized, may lead to inappropriate dose reductions, particularly for cancer chemotherapy or clozapine in the treatment of schizophrenia [46]. Invisibilization also occurs at the research level, where studies frequently exclude minority populations that do not fit the profile of the “ideal” patient, despite the fact that clinical reality more closely reflects these populations—for example, through the systematic exclusion of allophone patients in studies of migrant groups. From a transcultural medicine perspective, often mobilized as a second-line approach, attention to patients’ specific lived experiences may be clinically relevant, but only when it meaningfully contributes to the therapeutic relationship or the delivery of care, and when clinicians have the time and skills to do so [47,48]. Exploring patients’ representations of illness, treatment, and the health care system requires decentering and a deliberate avoidance of stereotyping, essentialization, or fascination, all of which constitute racist biases that are both harmful and detrimental to quality of care.

Second, medical education must explicitly deconstruct the use of racialized formulas or protocols based on weak or biased evidence [49], many of which originate in colonial or slave-based medical histories [50]. Across medical specialties, so-called race-based clinical adjustments highlight the need for stronger critical training on the historical and educational roots of medical knowledge [51-57] to prevent misdiagnosis, improve clinical reasoning, and emphasize that observed disparities are more plausibly related to chronic exposure to stress and trauma than to biological racial differences [58,59].

Finally, reframing racism as a public health risk factor—distinct from any notion of biological race [60,61]—“racism not race,” allows medical education to move beyond both culturalism and colorblindness [6]. This approach emphasizes structural, environmental, and psychosocial determinants of health [9], such as perceived discrimination, which has been shown to affect sleep [62], BMI [63], and engagement in high-risk behaviors [39], for example.

Patient safety is a term used repeatedly by participants and is an integral part of the explicit goal of combating health inequalities and discrimination in health care. The study includes some findings on how to achieve this, such as the need to include specific knowledge in teaching, while avoiding any cultural bias, and to teach about the psychological consequences of discrimination, which impact overall health. The more precise term “cultural safety,” raised by several participants, evokes the need to address the patient’s affiliations, namely their language, history, and perceptions. This term, prevalent in the literature [64,65] and tending to replace “cultural competence,” [66] is presented as a way to legitimize and value cultural differences in order to prevent harm in interactions between health care professionals and patients.

Clarifying the concept of race is therefore essential. As emphasized by scholars in political philosophy and psychiatry [37], race is not a matter of biological essence but of social assignment, stigmatization, and lived experience. Cultural affiliations are dynamic and shaped by shared histories, social positioning, and, in many cases, exposure to discrimination or trauma. According to researcher and psychiatrist Blanc [67], strengthening resilience and advancing health equity thus require holistic, multisectoral approaches that integrate historical, social, political, and educational dimensions alongside clinical care.

### Strengths and Limitations

A major strength of this study—uncommon in the French context—is the inclusion of diverse health professions educators’ perspectives on racism in health care within French-speaking settings. These findings identify concrete opportunities to develop structured educational interventions for medical students and to extend them to other health care professionals through a national network of educators sharing resources and pedagogical approaches.

This study focuses exclusively on educators’ perspectives and does not include those of students or patients. Although participants represented a range of professional backgrounds, regions, and countries of practice, future research should incorporate students’ and patients’ viewpoints to better assess the relevance, acceptability, and impact of these educational initiatives.

Another limitation is that because our inclusion criteria required prior publications and documented involvement in teaching initiatives, the sample overrepresents already-engaged educators and underrepresents those who are reluctant, resistant, or institutionally constrained, which may limit the diversity of perspectives captured. Also, nonspecialist teachers represent the majority of health professions educators. Future research should examine their roles and training needs, as their involvement will be essential to generalize this educational content.

Finally, this study may be influenced by social desirability and topic-engagement biases, as participants might have presented their views in ways they perceived as socially acceptable and were already invested in education on racial discrimination, potentially overrepresenting more positive or elaborated perspectives. In addition, given the small expert community and the detailed participant descriptors (profession and country), a risk of deductive disclosure cannot be fully excluded.

### Conclusion

Teaching about the effects of racial discrimination and racism on health aims to establish these issues as core public health concerns. Beyond the sense of medical powerlessness that may lead to the reliance on stereotypes within health care systems characterized by time constraints and pressure for rapid diagnosis and treatment, students must be supported in understanding how clinical practices are shaped by their historical and social embeddedness. In such contexts, patients from racialized minorities are at greater risk of reduced access to safe and equitable care.

The impact of racial discrimination on health must be explicitly addressed in medical education, as it is neither inevitable nor immutable, even though it is deeply intertwined with economic, political, and sociological processes that extend beyond the medical sphere. From a future perspective, the ongoing French debate regarding the feasibility of conducting robust epidemiological studies on racial health disparities remains unresolved. Rigorous epidemiological research on differential care represents a critical tool for making health disparities visible, identifying causal pathways, informing prevention strategies, and mobilizing policymakers. Such research also plays a key educational role by enhancing understanding of how discrimination can operate at every stage of care, from initial patient access to the full clinical encounter.

Raised by some participants, the issue of the place and experiences of health care workers who themselves come from ethnic minorities should be considered in future work.

## Supplementary material

10.2196/90084 Multimedia Appendix 1 Interview guides.

10.2196/90084 Checklist 1 COREQ reporting guidance.

## Abbreviations

COREQ: Consolidated Criteria for Reporting Qualitative Research
HSS: humanities and social sciences
INSERM: French National Institute for Health and Medical Research
TA: thematic analysis
