# Supplementary material for: Health and Humanities and Social Sciences Professionals’ Perceptions Regarding the Teaching of the Effects of Racism in Medicine: Semistructured Interview Study
Source: JMIR Med Educ. 2026 Apr 17;12:e90084. doi: 10.2196/90084 (PMC13089797; doi:10.2196/90084)
Supplement: Multimedia Appendix 1 [file mededu-v12-e90084-s001.docx]

**Appendices**

**Appendix 1: Interview Guide – Medical Professionals**

**1. Participant Introduction**

- Could you introduce yourself, including your current job title and educational background?
- Where have you worked throughout your career?

**2. Racial Discrimination and Healthcare**

- Do you consider racial discrimination in medicine to be an issue? If so, in what ways?
- Are you familiar with the term Mediterranean syndrome? When did you first encounter it?
- How can medical students be trained to handle situations involving racial discrimination in clinical settings?

**3. Medical Education**

- What recommendations would you make to improve how racial discrimination in mental or physical health is addressed in healthcare?
- How can we involve all students in this issue, not only those already aware of it?
- In your opinion, what is the most effective way to raise awareness among students?

**4. Teaching Formats and Medical Simulation**

- Which teaching formats have you found most effective in engaging students with these issues?
- In your view, what would be the ideal format, regardless of practical constraints?
- What does the concept of immersion evoke for you in the context of medical education addressing racial discrimination?
- Have you ever adapted immersion techniques for these issues, and what was your experience?

**Appendix 2: Interview Guide – Psychologists**

**1. Participant Introduction**

- Could you introduce yourself, including your current job title and educational background?
- Where have you worked throughout your career?

**2. Racial Discrimination and Healthcare**

- Do you consider racial discrimination in medicine to be an issue? If so, in what ways?
- Are you familiar with the term Mediterranean syndrome? When did you first encounter it?

**3. Psychological Tools**

- How would you explain to medical students or interns the psychological effects of discrimination?
- How can students and interns be trained to handle situations of racial discrimination in professional environments, where they may alternate between being students and doctors?
- In your opinion, what is the best way to raise awareness of personal biases?

**4. Psychological Awareness**

- What recommendations would you make to improve how racial discrimination in mental or physical health is addressed in healthcare?
- How can all students be involved in this issue, not just those already aware?
- What pathways are necessary to promote awareness?
- What psychological tools could be used to achieve this?

**Appendix 3: Interview Guide – Teacher-Researchers in Humanities and Social Sciences**

**1. Participant Introduction**

- Could you introduce yourself, including your current job title and educational background?
- Where have you worked throughout your career?

**2. Racial Discrimination and Healthcare**

- Do you consider racial discrimination in medicine to be an issue? If so, in what ways?
- Are you familiar with the term Mediterranean syndrome? When did you first encounter it?

**3. Use of Humanities and Social Sciences**

- How could the humanities and social sciences contribute to medical education?
- How should these disciplines be integrated into medical teaching?
- Can you share any experiences you’ve had in this area, and which formats do you think were most effective?

**4. Positioning of Students and Interns**

- From your perspective as a sociologist/historian/anthropologist, how can students and interns be trained to handle situations of racial discrimination in professional environments where they alternate between student and doctor roles?
- How can medical professionals best develop awareness of their own biases?
